# Impact of a structured sleep education program on mothers' knowledge and attitudes toward infant sleeping

**DOI:** 10.1016/j.heliyon.2024.e29885

**Published:** 2024-04-18

**Authors:** Sawsan Abuhammad, Alaa Bani Younis, Azza H. Ahmed

**Affiliations:** a Department of Maternal and Child Health, Faculty of Nursing, Jordan University of Science and Technology, Irbid, 22110, Jordan; b Purdue University School of Nursing, 502 North University Street, West Lafayette, IN 47907, United States

**Keywords:** Sleeping habits, Knowledge, Attitude, Infant

## Abstract

**Introduction:**

Sleeping is necessary for the infant growth and development. Sufficient and quality of sleep can have an impact on physical, cognitive, and emotional functioning. Infancy is a critical time for establishing healthy habits and routines. However, many infants were suffering from sleeping issues that impact their health.

**Objectives:**

This study aims to evaluate the effect of educational programs given to mothers regarding their infants' sleep on mothers' knowledge and attitudes toward infant's sleeping.

**Method:**

A quasi-experimental design for nonequivalent groups was used, and data was collected from 208 mothers with infants aged 5–12 months from all Jordanian governorates who had not been exposed to educational programs prior to this study. Data was collected in two stages: pre-test and post-test, with two weeks in between for both groups.

**Results:**

The final results indicated that the educational intervention had a significant impact on mothers' knowledge over time. It was found that mothers in the intervention group had significantly higher mean of infant sleep health knowledge at follow up time compared to their baseline time (B = 0.236, P 0.001). Also, the yielded analysis showed that there was no significant change in mothers' mean attitudes toward infants sleeping over time (P = 0.011). The mothers’ measured sleep health knowledge correlated positively and significantly statistically with their sleep health attitudes score (r = 0.436, P 0.010).

The infancy period, which spans from birth to approximately two years of age, is considered a precarious and influential phase in human development [1,2]. There are several causes why this period is considered highly important [3]. Infancy is a time of quick growth and development for the brain, marked by significant neurodevelopment, including the formation of neural connections and the development of brain structures. The brain's construction is highly elastic during infancy, making it highly accessible to experiences and environmental stimuli [4,5]. Infancy is a crucial time for sensory and motor development [5]. Infants are constantly exploring and interacting with their environment, which helps them develop their full range of senses (sight, hearing, taste perception, touch, and smell) and fine-tune their motor skills. This forms the foundation for later complex skills, such as language, social interaction, and problem-solving [6].

One of the factors that impact the overall health of the infant is sleeping. Sleeping is necessary and insufficient and poor quality of sleep can have a negative impact on physical, cognitive, and emotional functioning [[7], [8], [9], [10]]. Infancy is a critical time for establishing healthy habits and routines [11]. Mothers play a significant role in shaping their infants' sleep habits [12]. Most mothers are unable to provide enough knowledge when asked about their infant's sleep habits or other sleep-related issues [13]. Also, a high rate of sleep disorders and sleep disturbances are linked to both infant-related and mother-related daily life [14]. Amounts of sleep ‘have been reported to be related to mothers' attitudes toward their infants' health. Sleeping interventions usually aim to address pediatric sleep issues related to their culture and how mothers perceive sleep problems [15]. Research has recommended interventions to solve an infant's sleep problems should not be aimed exclusively at the infant, but also include an intervention to address the mother's worries about their infant's sleep [16]. Mothers need to be educated on biological characteristic changes in their infant's sleep [17,18]. They should also be made aware of the impact of their behaviors on their infant's sleep [19]. Owens, Palermo, and Rosen reviewed behavioral management approaches for infant sleep disorders. They concluded that to maintain the mother's positive effect on infant sleep, it's important to implement continuous mother intervention to reduce infant sleep disorders [20].

Addressing sleep issues require the establishment of interventions that include effective communication and involvement strategies as well as participation in intervention research to minimize the possibility of infant sleep disorders [21]. According to the International Classification of Sleep Disorders (ICSD), sleep disorders have been classified as: difficulty falling asleep without mother's interference; breathing sleep problems; excessive daytime sleep; delayed sleep awake rhythm; physiological events during sleep; sleep stereotypical movement; and else as late bedtime; fragmented sleep at night; sleep association with feeding, etc. [22].

Mothers with children at any developmental stage will have challenges, and this is also related to the infancy stage, which alters the family's daily activities [23]. Mothers report their infants' sleep changes in a considerable manner across the first year of life, with the major alteration in sleep problems occurring during the first six to 12 months of life, confirming an important link between infant sleep quality and maternal sleep quality [24].

A previous study from Jordan showed that traditional knowledge and cultural health attitudes often form the foundation for the practices used by mothers to care for their infants. This influence can disrupt any attempted mothers' intervention related to infant care issues [25]. A previous study from Jordan mothers reported that 45.1 % of the infants had anxious conditions and 52.3 % of the infants had good sleep habits. Education and intervention were reported to be efficient methods of assisting mothers with infant sleep issues [26]. According to a study in the Middle East, 37 % of mothers felt their infants had a sleep disorder. The mothers of the infants reported having a delayed sleep schedule. Consequently, going to bed late at night, waking up late in the morning, and resisting falling asleep are all independently predicted sleep disorder outcomes [27]. The need for sleep issues to be addressed by pediatric and adult healthcare is supported by the high prevalence of sleep problems in infants [27]. Limited studies have been conducted in the Middle East to discuss the impact of educational programs on knowledge, attitudes and sleeping habits in infants as they are the group most at risk for having sleeping disorders [27]. Providing mothers with strategies to manage their infants' sleep habits at an early will help them to modify their attitudes and to be more knowledgeable regarding their infants' sleep cues, problems, and sleep controlling. They will be better equipped to synchronize their infants' sleep with their own [28]. Furthermore, management of sleep disorders and establishing healthy sleep for infants with less fragmented sleep, less resistance to sleep, ability to fall asleep and self-soothe will reflect positive outcomes in the community by promoting healthy growth and normal developments for infants and minimizing the negative prolonged outcomes in the future that result from irregular sleep habits [29]. This study aims to evaluate the effect of educational programs given to mothers regarding their infants' sleep on mothers' knowledge and attitudes toward infant's sleeping.

## 1 Method

Convenience sampling was used to mothers who were able to select the group that they would like to participate in after explaining the study. Based on G power an adequate same size for nonequivalent group pre-post design, with a power level of 0,8 and significant alpha level 0,05 and moderate effect size of 0,25, was 80 participants at least for each group (160 for both groups). Eligibility criteria: a) All mothers in Jordan having infants with 5–12 months of age, b) mothers' age more than 18 years old, c) Able to speak and write in Arabic language. Mothers with infants who are unhealthy or exhibit congenital diseases or mood and behavioral disorders affecting their sleep patterns were excluded. This age range of infants were chosen because mothers of infants in this age group commonly encounter challenges related to sleep disturbances, making them receptive to guidance and support in improving their child's sleep patterns. Choosing this age range ensures that interventions are implemented during a critical period of sleep development while addressing the immediate needs of mothers seeking assistance with their infants' sleep behaviors.

### 1.1 Setting

The participants were recruited from all Jordanian governorates by using community setting (women charities and nurseries), and via social media announcements, such as Facebook, Instagram, WhatsApp, and other types of social media applications.

### 1.2 Survey instrument

#### 1.2.1 Demographic part

This part includes demographic information related to gender, age, religion, educational level, employment status for mothers, infants' sleeping duration, educational courses attended previously, having children with mood or behavioral and chronic disease diseases, and living place.

#### 1.2.2 Parents' knowledge and attitude survey

This instrument consists of a brief 2-page survey with 14 items developed by Judith Owens based on the principles of good sleep practices in children aged from 0 to 12 years [15]. These survey items addressed the following topics [1]: Basic Sleep Knowledge (10 items) which assess mothers' awareness related sleep issues that influence infants' growth and development and the necessity of sleep routine for infants. Initial version was piloted on 141 parents. The highest score is 10 and lowest score is 0. As the score increases, more knowledge regarding infant sleep. The Cronbach's alpha for the instrument is 0.87 [15]. The second part is attitude regarding sleep as a health behavior (4 items) which assess mothers' beliefs regarding healthy sleep of their infants and asked mothers to specify if their infant gets adequate sleep and has healthy sleep pattern, and if they plan to change their infant's sleep pattern or would like to talk to their infant's doctor regarding their sleep. The survey is Likert scale with scores ranging from strongly disagree to strongly agree. As the score increases, it means a more positive attitude toward infant sleep. The Cronbach alpha for this instrument is 0.88.

#### 1.2.3 Educational program (intervention)

Based on reviewing the literature regarding sleep among infants. The authors identified the best type of intervention to enhance mothers' knowledge, attitude, and practice toward optimal sleep patterns among infants. This education program that is given for mothers includes information that aims to enhance the mothers' knowledge and attitudes. It provides mothers tips and advice to deal with the bad sleeping habits for infants such as late bedtime, resistance to sleep, and fragments of sleep at night which results in improvement in infant sleeping pattern in a positive way.

This interventional program covers three significant infant sleeping problems and highlighted the issues that affect maintaining the healthy sleep pattern in positive way. Moreover, this program allows the mothers to be more able to recognize their infants sleep and assist them to regulate their sleep via synchronized baby sleep with internal clock (circadian clock), darkness hormone (melatonin) mechanism, and sleeping pressure (hemostasis). This program duration is about 5 h and could be given during a one-day session or scattered based on suitable time of the participants. The program was implemented through online platforms and occasionally through in-person sessions. In both modalities, presentations were conducted using PowerPoint slides in both Arabic and English languages. Educational materials for mothers were delivered via WhatsApp, Telegram, and Messenger applications.

#### 1.2.4 Data collection procedure

The participants who met the inclusion criteria were invited to participate in the study via social media groups; Facebook, Messenger, Instagram, Telegram and WhatsApp, also permission from head chair of child nurseries and women Charities was obtained to ask mothers to participate in the study. Moreover, phone numbers were given by these charities to the researcher to contact the groups of mothers. The data collection was completed between February 2023 and April 2023 by qualified nurses with a master's degree who received training about teaching this educational program. For the intervention group they fill the questionnaire prior to the program which takes around 5–7 min, then the program courses are given via online lectures, and face to face for some groups since the authors could not have the ability to collect them in the same place based on their preference and time. Then, after finishing the course the mothers could have the material of the session or the recorded session after their agreement via WhatsApp and telegram applications. After two weeks the participants in the intervention group were asked to fill in the questionnaire again for control group.

#### 1.2.5 Intervention group

All participants in the intervention group who chose to attend the program session were divided into small groups and participated in online or face to face groups as they preferred. The program session presented for the intervention groups according to time and application they prefer. All questionnaires filled by the participants pre session and two weeks post session (according to literature review the infants sleep pattern need around two weeks to be adjusted to new routine). During the session mothers had the opportunity to share their own problems regarding infant sleep. After finishing the session, PowerPoints material and records of lectures were sent to the participants via Telegram and What's up based on their preference.

### 1.3 Control group

All participants in the control group filled in the questionnaires twice. The first time was at the same time as the pretest of the intervention group. Then, they filled the same surveys with the same time of posttest of the interventional group. after finishing the second time of the questionnaires filling in the material sent to them via Telegram and WhatsApp to prevent any ethical dilemma.

#### 1.3.1 Ethical considerations

Ethical approval from the Institutional Review Board at Jordan University of Science and Technology was obtained before conducting the study. In addition, the ethical approval from the Ministry of health was obtained too. After having approval from the scientific research committee at the department of nursing and the Institutional Review Board (IRB) at 14/2023. The informed consent was filled from each participant. Each participant was provided with comprehensive details regarding the study's objectives, specific participation requirements—including time commitments—and any group preferred to be. Each group was presented with a tailored informed consent document. It was assured for the participants that there was no direct financial benefit or remuneration. The participants had the opportunity to select which group (control group or interventional group) they prefer to be in.

#### 1.3.2 Data analysis

The bivariate analysis methods were used to compare the two groups of mothers and infants on their sociodemographic factors, to ascertain that the two groups of mothers included into the study were equivalent with respect to their sociodemographic characteristics that may work as possible confounder variables for certain analyzed outcomes. The Chi-square test was used to compare the ratio of the categorical variables for demographic data between control and interventional groups. For the continuous variables of the independent group *t*-test was used.

## 2 Results

### 2.1 Demographical variables

A total of 208 mothers of infants aged between 5 and 12 months had enrolled in the study and completed and returned the study measured questionnaires. The average age for the included infants was 8.68 (SD = 2.13) months, and the youngest infants were aged 5 months and the eldest infants were aged 12 months. See Table 1.

**Table 1:** Bivariate comparison between the mother's groups on their sociodemographic characteristics.

	Control Group, n = 111	Intervention group, n = 97	test	P
**Infants Age (months), mean (SD)**	8.68 (2.11)	8.67 (2.16)	t = 0.34, df = 206	0.973
**Mother Age group**				
18–25 years	25 (22.5)	12 (12.4)	χ2 = 4.01/df = 2	0.132
26–35 years	72 (64.9)	68 (70.1)		
36–45 years	14 (12.6)	17 (17.5)		
**Mother's Educational Level**				
Secondary or less education	27 (14.3)	13 (13.4)	χ2 = 4.97/df = 2	0.083
University Degree	73 (65.8)	77 (79.4)		
Post-graduate	11 (9.9)	7 (7.2)		
**Mother's Occupational status**				
Unemployed	85 (76.6)	76 (78.4)	χ2 = 0.039/df = 1	0.760
Employed	26 (23.4)	21 (21.6)		

### 2.2 The impact of the education program on mothers' knowledge level of intervention groups

Cochran Q chi-squared test was used to compare mothers' knowledge levels from baseline-follow up times for each group separately. The results indicated that there was a significant difference among the mothers' study groups and their response in all items. The intervention group showed the overall mothers' knowledge level items had a significant difference between pre and post assessment (P < 0.001), except for item #6 (P = 0.366). The findings suggested a notable positive effect on maternal knowledge levels in intervention groups when mothers received an infant sleep education program. See Table 2.

**Table 2:** Bivariate chi-square test comparing mothers knowledge from before-after times for each group separately.

	(%)-correct answers			
	At baseline	*At follow up*	*χ2/df*^a^	P
CONTROL GROUP (N = 111)				
1) **Children who do not get enough sleep are more likely to be underweight than overweigh**t	9.9	***14.4***	*17.81/1*	<0.001
2) Snoring in a child indicates that he or she is sleeping well is not getting enough sleep	70.3	***75.7***	*0.974/1*	0.33
3) Being either under or overactive can both be warning signs that a child Is not getting enough sleep	45	***56.8***	*2.965/1*	0.085
4) **Watching TV in their bedroom makes it more difficult for children to fall asleep**	69.4	***72.1***	*0.184/1*	0.668
5) Children should have the same bedtime and wake time on weekdays and weekends	71.2	79.3	1.884/1	0.17
6) Children only need a bedtime routine if they are having trouble falling asleep	9.9	***17.1***	2.28/1	0.131
7) **Well-rested children do not need an alarm clock to wake up in the morning**	61.3	67.6	1.00/1	0.317
8) The average infants need about 10–12 h of sleep/24 h s	65.8	***68.5***	0.191/1	0.662
9) Being overweight can increase a child's risk of sleep problems	52.3	64.9	3.379/1	0.006
10) **Until children start school, they do not need a regular bedtime**	58.6	61.3	18.01/1	<0.001
**INTERVENTION GROUP (N** = **97)**				
1) **Children who do not get enough sleep are more likely to be underweight than overweight**	16.5	***45.4***	*17.82/1*	<0.001
2) Snoring in a child indicates that he or she is sleeping well is not getting enough sleep	66	***90.7***	*15.15/1*	<0.001
3) Being either under or overactive can both be warning signs that a child Is not getting enough sleep	42.3	***73.2***	*16.071/1*	<0.001
4) **Watching TV in their bedroom makes it more difficult for children to fall asleep**	69.1	***86.6***	*7.81/1*	0.005
5) Children should have the same bedtime and wake time on weekdays and weekends	74.2	92.8	10.81/1	0.001
6) Children only need a bedtime routine if they are having trouble falling asleep	4.1	***7.2***	0.818	0.366
7) **Well-rested children do not need an alarm clock to wake up in the morning**	60.8	89.7	20.63/1	<0.001
8) The average infants need about 10–12 h of sleep/24 h s	58.8	89.7	22.5/1	<0.001
9) Being overweight can increase a child's risk of sleep problems	47.4	78.4	18.00/1	<0.001
10) **Until children start school, they do not need a regular bedtime**	61.9	86.6	13.71/1	<0.001

Note: The Cochran's Q chi-squared test compares the proportions of before and after measured outcomes for a significant change, any change a decrease or increase or no change alike.

a ***χ2-test For repeated binary measures.***

### 2.3 The impact of education program on mothers' attitude of interventional groups

The *t*-test assessed the differences in the infants' measured sleep habits between the baseline and follow-up times. A separate analysis was used for the mothers’ groups (control vs intervention groups) for the total score and for item by item. Specifically, in the control group the post assessment of mothers' attitudes showed an insignificant change from the pre-assessment. On the other hand, in the intervention group, the overall mothers' attitudes items had a significant difference between pre- and post-assessment (P < 0.001). The final result indicates that there is a significant positive impact on their attitudes when an infants' sleep education program is given to mothers. See Table 3.

**Table 3:** Difference in attitude among mothers before and after having educational program mean (SD).

	Baseline	Follow up	t/df	p
Intervention Group				
1. I believe my child gets enough sleep	4.10 (2.04)	5.59 (1.48)	2.52/97	<0.001
2. I believe my child has healthy sleep habits	3.90 (2.01)	5.39 (1.52)	2.30/97	<0.001
3 I plan to change my child's sleep habits (R)	4.61 (2.37)	2.75 (1.96)	0.89/97	<0.001
4. would like to talk to my child's doctor about his/her sleep (R)	4.05 (2.39)	2.33 (1.84)	3.1/97	<0.001
**Control Group**				
1. I believe my child gets enough sleep	4.20 (2.04)	4.40 (2.14)	2.12/111	0.17
2. I believe my child has healthy sleep habits	3.33 (2.01)	3.83 (2.11)	2.40/111	0.131
3 I plan to change my child's sleep habits (R)	4.67 (2.37)	4.22 (2.37)	0.82/111	0.317
4. would like to talk to my child's doctor about his/her sleep (R)	4.23 (2.39)	4.33 (2.19)	3.54/111	0.662

### 2.4 The combined and individual effects of the educational program on mothers' knowledge regarding infant sleeping

To assess the combined and individual effects of the educational program on the mothers' knowledge level over time and to find the outcomes of mothers' repeated mean measure of knowledge level scores at baseline and follow-up times, the generalized linear mixed models (GL Mixed) analysis was used. In this model we tried all relevant predictors based on literatures, some of the variables below were not significant but included in the model to increase the accuracy. The findings showed that the mother's age did not impact their knowledge level regarding infants' sleep throughout the study time (both at baseline and follow-up time). Also, the mother's employment did not impact their infants' sleep health knowledge (P = 0.769). The analysis model showed that the intervention group of mothers and the control group of mothers did not differ significantly on their mean infants knowledge level scores at baseline time, but the analysis model showed that the effect of the educational intervention over time had a significant impact on mothers' knowledge level. It was found that mothers in the intervention group had a significantly higher mean of their knowledge level regarding infants' sleep habits at follow-up time compared to their baseline time (B = 0.236, P < 0.001), while the mothers in the control group did not show a significant increase in their knowledge level regarding infants' sleep habits at follow-up compared to their baseline time (P < 0.00.220). Moreover, the multivariable analysis model showed that the mothers' mean perceived infants' sleep health attitude score had correlated significantly and positively with their mean measured knowledge score throughout the study period (B = 0.018, P < 0.001). Mothers with greater infants' sleep health attitudes showed significantly higher infants' sleep knowledge score in general. The mother and infant measured factors and predictors did not correlate significantly with the mothers' sleep health knowledge score when tested in the models conducted by the researchers, therefore were dismissed from the analysis model. See Table 4.

**Table 4:** Multivariable Generalized Linear Mixed Model explaining the mother's knowledge score throughout the study period.

Predictor	95 Confidence Interval for (β)			
	Beta (β) coefficient	Lower	Upper	P
Intercept	1.229	1.041	1.418	**<0.001**
Mother age = 36–45 yrs.	0.061	−0.096	0.218	0.446
Mother age = 26–35 yrs.	0.007	−0.101	0.114	0.901
Employed mother	0.017	−0.096	0.129	0.769
Treatment Group = Intervention	−0.001	−0.117	0.115	0.988
Interaction: Time* Treatment = Follow up time*Intervention group	0.236	0.138	0.334	**<0.001**
Interaction: Time* Treatment = Follow up time*control group	0.059	0.036	0.155	**0.220**
Mother's mean perceived sleep health attitude score	0.018	0.011	0.024	**<0.001**

**Dependent outcome variable** = **Mother's sleep health knowledge score.**

### 2.5 The combined and individual effects of the educational program on mothers' attitudes score regarding infants’ sleep

To assess the impact of the intervention program on mothers' mean attitudes score throughout the study, the mothers' repeated mean score was subjected to the multivariable Generalized Linear Mixed Models analysis. The findings showed that mothers in the educational intervention group did not differ significantly with respect to their baseline attitudes compared to mothers in the control group (P = 0.781), denoting that both groups of mothers measured nearly equivalent attitudes scores at baseline time before the intervention. Also, the yielded analysis model showed that there was no significant change in mothers' mean attitudes toward infants' sleep through time (P = 0.011). The two groups of mothers exhibited significant differences in their attitudes measured over time, with an insignificant interaction observed between the educational intervention and time (Time*treatment, P = 0.794). This suggests that although mothers in the intervention group showed a slight increase in their mean attitude scores at follow-up compared to baseline, this increase did not significantly differ from that observed in the control group. Also, the mother's age (in years) did not correlate significantly with their mean perceived attitudes toward infants' sleep habits (P = 0.755). The yielded analysis model findings indicated that the mean See Table 5.

**Table 5:** Multivariable Generalized Linear Mixed Model Regression explaining the mother's mean perceived sleep health attitudes score. N = 416.

Predictor	95 CI for beta (β) coefficient			
	Beta coefficient	Lower	Upper	P
Intercept	2.868	2.693	3.043	<0.001
Treatment Group = Intervention	0.012	−0.07	0.094	0.781
Time = Follow up time	0.102	0.024	0.18	0.011
Interaction (time*treatment = Follow up time*Intervention)	0.017	−0.108	0.141	0.794
Mother's age in years	−0.008	−0.057	0.042	0.755
Infants CSHQ total score (infants problematic sleep score)	−0.006	−0.009	−0.003	<0.001
Mother's Sleep Health knowledge score	0.06	0.046	0.075	<0.001

*Dependent outcome variable* = *Mother's mean perceived sleep health attitudes score*.

## 3 Discussion

The results of our study showed that educational intervention about infants' sleep habits had a significant positive impact on mothers' knowledge level of the issues over time. Moreover, the mothers in the intervention group showed a significant improvement of their knowledge at follow-up time in establishing bedtime routines for children to enhance their sleep. In general, the intervention group showed the overall mothers' knowledge items had a significant difference between pre- and post-assessment (P < 0.001), except for item #6 (P = 0.366). This was consistent with the results of previous studies. In Egypt, Ibrahim, Sobeh and Zaghamir [30] showed a significant positive impact on maternal knowledge regarding infant sleep habits. Similarly, a review carried out by Mihelic et al. [31] in the USA showed that educational interventions are important to improve maternal knowledge regarding infants' sleep habits. Also, Oliveira et al. [32] in Brazil, showed that maternal knowledge was improved after educational interventions. It should be noted that there are no previous studies in the literature review that disputed the results of our study. The intervention of our study was effective and had a positive impact that improved mothers' knowledge regarding infants' sleep habits.

Our findings showed that the educational intervention on infants' sleep habits had no significant positive impact on mothers' attitudes regarding their infants' sleep habits. The study findings indicate that, regarding mothers' opinions about their infants having healthy sleep habits, getting enough sleep, planning to change sleep habits, and consulting their doctor about infants' sleep habits, there is no significant difference between pre- and post-assessment (P < 0.781). The mothers' attitudes did not change over time, a finding which is not consistent with those of the previous studies. Mihelic et al. [31] in the USA and Rafihi-Ferreira et al. [33] in Brazil both found that educational interventions are important to improving attitudes about infants' sleep. Similarly, in Iceland, Sigurdardottir and Svavarsdottir [34] carried out a study that showed that educational programs improve maternal attitudes regarding perceived infants' sleeping habits. In summary, education programs given to mothers related to infant sleep habits have no significant impacts over time to improve their attitude toward their infants' sleep habits. This may point to the fact that more time is needed to change mothers' attitudes. In general, that indicates the need for more research to study mothers' attitudes toward their infants’ sleep habits over time as it very difficult to change human attitudes.

Our findings showed that socio-demographic characteristics of the mothers (age of mothers and infants, employment status and education level) have no significance on their knowledge level regarding their infants' sleep habits throughout the study time (both at baseline and follow-up time). However, the interaction effect of educational intervention with time had a significant impact on mothers' knowledge level over time. Moreover, our study results showed that mothers' attitudes correlated positively and significantly with mothers' knowledge levels. This result is consistent with previous studies. For example, the systemic reviews carried out by Landsem [35]in Norway and McDowall et al. [36] in New Zealand showed that knowledge about infant sleep is very complicated with multiple factor effects and mothers who received education about infant sleep were more expected to describe their infants' sleep habits. In contrast, Lupini et al. [37] and Zanetti et al. [38] showed that race, family context, socioeconomic and environmental structure impact on maternal knowledge levels. On the other hand, Herman et al. [39] and the systematic review by Covington et al. [40] both concluded that there are many predictors of knowledge toward infants’ sleeping habits. These include own belief, influence of family and friends, and minimal education. Thus, the final result of our study confirms that maternal knowledge regarding infant sleep is influenced by mothers' attitudes and intervention effect.

The findings showed that neither for the two mothers' groups (control and intervention) differ significantly with respect to their follow-up time reported attitude as evidenced by an insignificant interaction term between the educational intervention and time (Time*treatment). This indicates that, despite the fact the mothers in the intervention group measured a slight increase in their mean attitude score at follow-up time compared to baseline time, the increase in their mean attitude score did not differ significantly from the mothers in the control group where no educational intervention was implemented. However, the mother's age (in years) did not correlate significantly with their mean perceived attitudes toward infants' sleep health. In the UK, Rudzik and Ball [41] and McClellan [42]showed the attitudes of mothers affect healthy infant sleep. Also, maternal attitude influences their infant's sleep more than their own knowledge.

### 3.1 Implications

Nursing educators should educate student nurses about the importance of mothers' education regarding their infants' sleep. The care of infants is family-centered; thus it is crucial that mothers be educated on how to handle sleep problems in order to avoid the possible future harms related to growth and development consequences for infants. Also, students should be trained on how to conduct the educational program that was used in this study so they can, in turn, enable mothers to use this intervention in their lives to improve their infants’ sleep habits.

### 3.2 Strengths and limitations

The advantage of this study is being a national study focusing on the infant population in the Middle-east country that have sufficient subjects. On the other hand. One of the limitations of this study is delaying in having the agreement to use the tool at the time of data collection from the original author which impact the study. Moreover, there were issues to collect the data at the same time because of the convenience of the participants for the time and location since the data collected from all over Jordan. Another problem is the generalizability of the finding on other countries since it is just conducted in Jordan with different culture.

## 4 Conclusion

This study has shown that mothers' knowledge and attitudes can be enhanced through an education program which also establishes healthy sleep habits for infants. Moreover, the mother knowledge and attitude toward sleeping for infants are correlated positively with each other. This study could help to translate the results of this study to the health care system.

## Funding

This work was supported by Deanship of Research, 10.13039/501100004035Jordan University of Science and Technology # 20230123.

## Data availability

Data will be available upon reasonable request from the author.

## CRediT authorship contribution statement

**Sawsan Abuhammad:** Writing – review & editing, Writing – original draft, Visualization, Validation, Supervision, Resources, Methodology, Investigation, Formal analysis, Data curation, Conceptualization. **Alaa Bani Younes:** Writing – review & editing, Writing – original draft, Validation, Software, Project administration, Investigation, Funding acquisition, Data curation. **Azza H. Ahmed:** Writing – review & editing, Writing – original draft, Visualization, Validation, Supervision, Resources, Project administration, Investigation, Funding acquisition, Formal analysis, Conceptualization.

## Declaration of competing interest

The authors declare that they have no known competing financial interests or personal relationships that could have appeared to influence the work reported in this paper.
