# Supplementary material for: Impact of a structured sleep education program on mothers' knowledge and attitudes toward infant sleeping
Source: Heliyon. 2024 Apr 18;10(9):e29885. doi: 10.1016/j.heliyon.2024.e29885 (PMC11070819; doi:10.1016/j.heliyon.2024.e29885)
Supplement: Multimedia component 1 [file mmc1.docx]

**Child' Sleep Habits Questionnair- Infants Version (CSHQ-I)**

| **Child sleep habits questionnaires- infant version**  **استبيانات عادات نوم الطفل - نسخة الرضع** |
| --- |

| **not applicable**  **غير مطبق** | **Rare**  **نادراً** | **Sometimes**  **أحيانا ً** | **Usually**  **عادة** |  |
| --- | --- | --- | --- | --- |
| **مقاومة الطفل للنوم** | | | | **Sleep time resistance** |
|  |  |  |  | 1. **Child falls asleep within 20 min after going to bed**   **ينام الطفل في غضون 20 دقيقة بعد النوم** |
|  |  |  |  | 1. **Child falls asleep alone in own bed**   **ينام الطفل وحده في سريره** |
|  |  |  |  | 1. **Child falls asleep with rocking or rhythmic movement**   **ينام الطفل بحركة هزازة أو إيقاعية** |
|  |  |  |  | 1. **Child needs parent in the room to fall asleep**   **يحتاج الطفل أحد الوالدين في الغرفة ليغفو** |
|  |  |  |  | 1. **Child resists going to bed at bedtime**   **يقاوم الطفل الذهاب للنوم وقت النوم** |
|  |  |  |  | 1. **Child struggles at bedtime (cries, refuses to stay in bed, etc.)**   **يكافح الطفل وقت النوم (يبكي ، يرفض البقاء في السرير ، إلخ(** |
|  |  |  |  | 1. **Child sleeps too little**   **ينام الطفل قليل جدا** |
|  |  |  |  | 1. **Child sleeps the right amount**   **الطفل ينام القدر المناسب** |
|  |  |  |  | 1. **Child moves to someone else’s bed during the night (parent, brother, sister, etc.)**   **ينتقل الطفل إلى سرير شخص آخر أثناء الليل (الوالد ، الأخ ، الأخت ، إلخ(.** |
|  |  |  |  | 1. **Child awakes more than once during the night.**   **الطفل يستيقظ أكثر من مرة أثناء الليل** |
|  |  |  |  | 1. **Child returns to sleep without help after waking.**   **يعود الطفل للنوم دون مساعدة بعد الاستيقاظ** |
|  |  |  |  | 1. **Child wakes up very early in the morning.**   **الطفل يستيقظ مبكرا جدا في الصباح.** |
| **Sleep Anxiety : قلق النوم** | | | | |
|  |  |  |  | 1. **Child is afraid of sleeping in the dark.**   **يخاف الطفل من النوم في الظلام** |
|  |  |  |  | 1. **Child is afraid of sleeping alone.**   **Child is restless and moves a lot during sleep**  **الطفل يخاف من النوم وحده الطفل قلق ويتحرك كثيرًا أثناء النوم** |
|  |  |  |  | 1. **Child snores loudly.**   **Child seems to stop breathing during sleep.**  **الطفل يشخر بصوت عال يبدو أن الطفل يتوقف عن التنفس أثناء النوم** |
|  |  |  |  | 1. **Child has trouble sleeping away from home (visiting relatives, vacation).**   **يجد الطفل صعوبة في النوم بعيدًا عن المنزل )زيارة الأقارب ، الإجازة(** |
|  |  |  |  | 1. **Child awakens during night screaming, sweating, and inconsolable.**   **يستيقظ الطفل أثناء الليل وهو يصرخ ويتعرق ولا عزاء له** |
|  |  |  |  | 1. **Child awakens alarmed by a frightening dream.**   **يستيقظ الطفل منزعجًا من حلم مخيف** |
|  |  |  |  | 1. **Child wakes up in negative mood.**   **الطفل يستيقظ في مزاج سلبي** |
|  |  |  |  | 1. **Child seems tired.**   **يبدو الطفل متعبا** |
| **Positive Sleep Habits عادات نوم ايجابية** | | | | |
|  |  |  |  | 1. **Child goes to bed at the same time at night.**   **يذهب الطفل إلى الفراش في نفس الوقت ليلا** |
|  |  |  |  | 1. **Child needs special object to fall asleep (doll, special blanket, etc.)**   **يحتاج الطفل إلى شيء خاص لينام )دمية ، بطانية خاصة ، إلخ(.** |
|  |  |  |  | 1. **Child is ready to go bed at bedtime.**   **الطفل مستعد للذهاب للنوم في وقت النوم** |
|  |  |  |  | 1. **Child sleeps around the same amount each day.**   **ينام الطفل بنفس المقدار من النوم كل يوم** |
|  |  |  |  | 1. **Child wakes up by himself.**   **الطفل يستيقظ من تلقاء نفسه** |
|  |  |  |  | 1. **Child has a good appetite in the morning.**   **الطفل يستقيظ بشهية جيدة للطعام** |
| **Daytime Sleepiness النعاس أثناء النهار** | | | | |
|  |  |  |  | 1. **Child falls asleep in parent’s or sibling’s bed.**   **ينام الطفل في سرير أحد الوالدين أو الأشقاء** |
|  |  |  |  | 1. **Child grinds teeth during sleep (your dentist may have told you this).**   **يطحن الطفل أسنانه أثناء النوم )ربما أخبرك طبيب أسنانك بذلك(** |
|  |  |  |  | 1. **Adults or siblings wake child up.**   **يستيقظ الكبار أو الأشقاء** |
|  |  |  |  | 1. **Child has difficulty getting out of bed in the morning.**   **يجد الطفل صعوبة في النهوض من الفراش في الصباح** |
|  |  |  |  | 1. **Child takes a long time to become alert in the morning.**   **يستغرق الطفل وقتًا طويلاً حتى يصبح يقظًا في الصباح** |
